# Common and distinct cortical thickness alterations in youth with autism spectrum disorder and attention-deficit/hyperactivity disorder

**DOI:** 10.1186/s12916-024-03313-2

**Published:** 2024-03-04

**Authors:** Wanfang You, Qian Li, Lizhou Chen, Ning He, Yuanyuan Li, Fenghua Long, Yaxuan Wang, Yufei Chen, Robert K. McNamara, John A. Sweeney, Melissa P. DelBello, Qiyong Gong, Fei Li

**Affiliations:** 1 Department of Radiology and Huaxi MR Research Center (HMRRC), Functional and Molecular Lmaging Key Laboratory of Sichuan Province, West China Hospital, Sichuan University, Chengdu, 610041 Sichuan People’s Republic of China; 2 Research Unit of Psychoradiology, Chinese Academy of Medical Sciences, Chengdu, 610041 Sichuan People’s Republic of China; 3 Department of Radiology, Sir Run Run Shaw Hospital, Zhejiang University School of Medicine, Hangzhou, 310016 Zhejiang People’s Republic of China; 4 Department of Psychiatry, West China Hospital, Sichuan University, Chengdu, 610041 Sichuan People’s Republic of China; 5 Department of Psychiatry and Behavioral Neuroscience, University of Cincinnati, Cincinnati, OH 45219 USA

**Keywords:** Autism spectrum disorder, Attention-deficit/hyperactivity disorder, Cortical thickness, Meta-analysis, Magnetic resonance imaging, Surface-based morphometry

## Abstract

**Background:**

Autism spectrum disorder (ASD) and attention-deficit/hyperactivity disorder (ADHD) are neurodevelopmental disorders with overlapping behavioral features and genetic etiology. While brain cortical thickness (CTh) alterations have been reported in ASD and ADHD separately, the degree to which ASD and ADHD are associated with common and distinct patterns of CTh changes is unclear.

**Methods:**

We searched PubMed, Web of Science, Embase, and Science Direct from inception to 8 December 2023 and included studies of cortical thickness comparing youth (age less than 18) with ASD or ADHD with typically developing controls (TDC). We conducted a comparative meta-analysis of vertex-based studies to identify common and distinct CTh alterations in ASD and ADHD.

**Results:**

Twelve ASD datasets involving 458 individuals with ASD and 10 ADHD datasets involving 383 individuals with ADHD were included in the analysis. Compared to TDC, ASD showed increased CTh in bilateral superior frontal gyrus, left middle temporal gyrus, and right superior parietal lobule (SPL) and decreased CTh in right temporoparietal junction (TPJ). ADHD showed decreased CTh in bilateral precentral gyri, right postcentral gyrus, and right TPJ relative to TDC. Conjunction analysis showed both disorders shared reduced TPJ CTh located in default mode network (DMN). Comparative analyses indicated ASD had greater CTh in right SPL and TPJ located in dorsal attention network and thinner CTh in right TPJ located in ventral attention network than ADHD.

**Conclusions:**

These results suggest shared thinner TPJ located in DMN is an overlapping neurobiological feature of ASD and ADHD. This alteration together with SPL alterations might be related to altered biological motion processing in ASD, while abnormalities in sensorimotor systems may contribute to behavioral control problems in ADHD. The disorder-specific thinner TPJ located in disparate attention networks provides novel insight into distinct symptoms of attentional deficits associated with the two neurodevelopmental disorders.

**Trial registration:**

PROSPERO CRD42022370620. Registered on November 9, 2022.

**Supplementary Information:**

The online version contains supplementary material available at 10.1186/s12916-024-03313-2.

## Background

Autism spectrum disorder (ASD) and attention-deficit/hyperactivity disorder (ADHD) are prevalent neurodevelopmental disorders in children and adolescents. ASD is characterized by social impairments, communication deficits, restricted interests, and stereotypical repetitive behaviors, while ADHD is defined by inattention, hyperactivity, and impulsivity [1]. Although different in many respects, it has been widely recognized that ASD and ADHD have overlapping behavioral features [2, 3] and genetic liability [4]. Specifically, social impairment and attention deficits are implicated in both disorders [2], and there are shared difficulties in other cognitive and behavioral traits [3]. Exploring brain structure could help understand the neurobiological basis which builds the bridge between the shared and different clinical manifestations and genetic liability of the two disorders. Currently, the overlapping and distinct brain mechanisms contributing to these two disorders remain to be clarified.

Previous structural magnetic resonance imaging (MRI) studies have revealed brain alterations in both ASD and ADHD [5–8]. Relative to typically developing controls (TDC), studies of ASD have demonstrated multiple regional morphological changes, some of which have been associated with social and behavioral features of autism [6]. In one study, individuals with ASD showed gray matter concentration increases prominently in the frontal, parietal, and occipital lobes, as well as subcortical regions, and decreases were observed in the temporoparietal junction (TPJ) [9]. Several studies of ADHD indicate that this disorder as well may result from delayed brain maturation, with delayed maturation of cortical volume, thickness, and surface area in pediatric patients with ADHD compared to TDC [7, 10, 11]. More recently, neuroimaging studies have begun to directly compare patients with ASD, ADHD, and comorbid individuals, and the findings of such studies have been inconsistent [12]. For example, overlapping abnormalities of reduced gray matter volume (GMV) in the left temporal lobe were seen in both ASD and ADHD [13], while another study found that enlarged GMV in left temporal cortex only in ASD [14]. These and other inconsistencies may be due to small samples with clinical heterogeneities [13, 14]. Therefore, a meta-analytic approach is well-suited to identify the most replicable overlapping and disorder-specific brain alterations in these disorders.

There have been previous meta-analytic efforts to compare alterations of brain anatomy in ASD and ADHD. A voxel-based meta-analysis of volumetric measurements reported increased bilateral temporal and right dorsolateral prefrontal volume in ASD and decreased ventromedial orbitofrontal volume for ADHD [15]. Another meta-analysis did not find significant differences in brain gyrification between the two disorders or between each disorder and TDC [16]. Cortical thickness (CTh) is a sensitive metric for evaluating cortical maturation abnormalities in clinical populations [17, 18]. Of note, it is sensitive to alterations in the maturation in the columnar organization of the neocortical mantle. Also, as regional volume measurements reflect combined influences of cortical morphology, examining CTh separately may advance neurobiological understanding of neurodevelopmental disorders [19]. ENIGMA consortium has found subtle overlapping cortical thinning in precentral gyrus and temporal lobes between the two disorders in children [20]. This study recruited data from multi-consortium sites rather than summarizing the data from existing publications and analyzed the average CTh in 68 cortical regions defined by the Desikan–Killiany atlas. The whole-brain vertex-based analysis might better address the issue of atlas bias in findings and report between-group differences in a more accurate brain location.

Another important issue for previous meta-analyses is that they included individuals with wide-ranging age groups [15, 16, 21]. For example, patients aged from less than 10 to over 60 in one study [21]. This is a potential limitation as structural abnormalities vary at different ages in both ASD [6] and ADHD [7], with children and adolescents having more significant atypicality than adults [22]. For example, atypicality of frontal, occipital, and parietal cortical volumes has been shown to be greater in adolescents than in adults with ASD [22]. Therefore, exploring brain features in children and adolescents may be more sensitive to detect neurodevelopmental alterations in brain maturation in ASD and ADHD, and their similarities and differences.

For these reasons, we conducted a whole-brain vertex-based CTh meta-analysis, with CTh measured as the distance between the gray-white interface and the pia mater. A recently developed mask for surface-based meta-analysis was used, which has been used previously in studies of other neuropsychiatric disorders [23]. To identify shared and disorder-specific CTh abnormalities in ASD and ADHD, a quantitative, vertex-based meta-analytic comparison of published whole-brain structural MRI studies in children and adolescents with ASD and ADHD was performed.

## Methods

### Search strategy and study inclusion

The present study was conducted according to the Preferred Reporting Items for Systematic Reviews and Meta-Analyses (PRISMA) guidelines (see Additional file 1: Table S1). A systematic search was conducted for peer-reviewed English language publications in PubMed, Web of Science, Embase, and Science Direct from inception to 8 December 2023. Keywords related to ASD (“autism” or “autistic” or “ASD” or “autism spectrum disorder”) and ADHD (“hyperkinetic” or “ADHD” or “attention-deficit/hyperactivity disorder”) plus terms associated with structural imaging (“cortical thickness” or “thickness”) were used for the literature search. A manual search was further conducted in the bibliographies of the retrieved studies and relevant reviews or meta-analyses.

The inclusion criteria for eligible studies included: (1) all participants were younger than 18 years of age and compared CTh between either of ASD and ADHD groups and TDC, (2) applied vertex-based or surface-based method, (3) estimated whole-brain CTh changes to remove bias inherent in regions-of-interest analysis, and (4) provided the peak coordinates of results in stereotactic space (Montreal Neurological Institute (MNI) or Talairach). We focused on the vertex-level whole-brain studies and excluded template-based studies even if the template covered the whole brain to reduce methodological heterogeneity and improve the spatial accuracy of results. Studies containing multiple independently analyzed subgroups were treated as separate datasets. For studies with multiple publications using overlapping samples, the one with the largest sample was included. Conference papers, case reports, and mega-analyses were excluded. Eligible studies reporting no between-group differences were included and estimated conservatively to have a null effect size. Studies were independently ascertained by two researchers (WFY and LZC) and checked by the corresponding author (FL). Any inconsistency was discussed under FL’s guidance until a consensus was reached. The protocol (registration number: CRD42022370620) was registered in the international prospective register of systematic reviews (PROSPERO).

### Quality assessment

There were four ASD studies [24–27] and two ADHD studies [28, 29] that could not be included due to unavailable coordinates of the cortical thickness (CTh) results after the corresponding authors were contacted for missing information (Additional file 1: Table S2). We applied the 12-point checklist to assess methodology quality of the included studies (Additional file 1: Table S3). In the 12-point checklist, each point was scored as 0, 0.5, or 1 if the criteria were unfulfilled, partially met, or fully met, respectively. All studies included in the present meta-analysis scored more than eight points. The checklist was not designed to critique the investigators or the work itself, but to provide an objective indication of the rigor of the individual studies. All studies using public databases were listed in Additional file 1: Table S4.

Mean age, mean IQ, proportion of males, comorbidity, medication status, preprocessing method, statistical threshold, and key findings of each study were summarized (Table 1). Effect size and coordinates of peaks for regional differences were also extracted for meta-analysis. Two co-authors (WFY and LZC) independently conducted the data extraction and the corresponding author (FL) double-checked the information.

**Table 1:** Clinical characteristics and summary findings of these ASD or ADHD studies included in this meta-analysis

Study	Dataset	Patients					Typically developing controls			Methods			Summary findings
		Number (male)	Mean age (SD), years	Mean IQ (SD)	Comorbidity	Medication status (%)^a^	Number (male)	Mean age (SD), years	Mean IQ (SD)	Preprocessing	Statistical threshold	Field strength	
ASD studies													
Raznahan et al. (2013) [30]		66 (66)	3.8 (1.0)	NA	NA	NA	29 (29)	3.8 (1.1)	NA	CIVET	*p* < 0.05 (corrected)	1.5 T	ASD > TDC: L SFG, superior temporal sulcus; R SFG, MFG, superior temporal sulcus, rostral intraparietal sulcus
Duerden et al. (2013) [31]		33 (29)	10.7 (2.5)	104.0 (18.3)	NA	Medication-free	30 (26)	10.7 (2.5)	113.6 (13.7)	CIVET	*p* < 0.05 (corrected)	1.5 T	-
Schaer et al. (2013) [32]		11 (8)	12.9 (2.7)	79.4 (18.1)	NA	NA	11 (8)	12.7 (2.7)	110.5 (13.3)	Freesurfer	*p* < 0.05 (corrected)	3.0 T	-
Dierker et al. (2015) [33]		34 (28)	11.4 (1.9)	111.5 (12.5)	NA	NA	32 (23)	11.3 (1.8)	114.9 (11.3)	Freesurfer	*p* < 0.025 (corrected)	3.0 T	-
Foster et al. (2015) [9]		38 (38)	12.4 (2.4)	102.5 (17.0)	Pure	NA	46 (46)	12.6 (2.6)	113.1 (12.0)	CIVET	*p* < 0.001 (uncorrected)	3.0 T	ASD > TDC: L IFG; ASD < TDC: R angular gyrus
Sussman et al. (2015) [34]		72 (61)	NA	NA	NA	NA	138 (116)	NA	NA	CIVET	*p* < 0.05 (corrected)	3.0 T	-
Yang et al. (2016) [35]		60 (60)	8.4 (2.1)	103.1 (14.5)	NA	NA	41 (41)	8.8 (2.3)	107.0 (15.0)	Freesurfer	*p* < 0.05 (corrected)	3.0 T	-
Tanigawa et al. (2018) [36]		16 (16)	13.4 (1.1)	112.7 (13.8)	Pure	NA	17 (17)	13.4 (1.2)	118.4 (15.0)	Freesurfer	*p* < 0.01 (corrected)	3.0 T	-
Kohli et al. (2019) [37]	Data from SDSU	64 (52)	13.3 (2.7)	106.7 (16.9)	NA	NA	64 (55)	13.5 (3.0)	106.5 (14.1)	Freesurfer	*p* < 0.01 (corrected)	3.0 T	ASD < TDC: R insula
	Data from NYU	31 (31)	11.4 (2.8)	103.2 (10.1)	NA	NA	31 (31)	11.8 (2.6)	110.5 (11.9)	Freesurfer	*p* < 0.01 (corrected)	3.0 T	-
Yin et al. (2022) [38]	Data from SI	18 (12)	12.0 (3.5)	99.9 (18.8)	ADHD, anxiety disorder	NA	18 (12)	12.7 (NA)	NA	Freesurfer	*p* < 0.05 (corrected)	1.5 T	-
	Data from RU	15 (12)	12.7 (4.6)	100.9 (14.4)	ADHD, anxiety disorder	NA	15 (8)	10.9 (NA)	NA	Freesurfer	*p* < 0.05 (corrected)	3.0 T	-
ADHD studies													
Qiu et al. (2011) [39]		15 (15)	12.7 (1.8)	NA	Pure	Medication-free	15 (15)	13.2 (1.7)	NA	Freesurfer	*p* < 0.05 (uncorrected)	3.0 T	ADHD < TDC: R frontal lobe, L frontal lobe, R cingulate cortex
de Zeeuw et al. (2012) [40]		99 (87)	10.5 (2.0)	101.6 (16.0)	ODD, CD	Medication-free	101 (85)	10.1 (1.8)	106.0 (12.9)	CLASP^b^	*p* < 0.05 (corrected)	1.5 T	-
Hoekzema et al. (2012) [41]		43 (35)	11.6 (2.9)	NA	Pure	Medicated (88%)	41 (28)	11.2 (3.0)	NA	Freesurfer	*p* < 0.05 (corrected)	1.5 T	ADHD < TDC: L IPL, lingual gyrus, pre-CG, OFG; R pre-CG, OFG, IPL
Saute et al. (2014) [42]		18 (11)	12.1 (3.2)	93.4 (9.2)	Epilepsy	Medicated (39%)	46 (19)	13.1 (3.3)	110.2 (11.2)	Freesurfer	*p* < 0.05 (corrected)	1.5 T	ADHD < TDC: L SPL, para-CG, insula; R IPL, pars opercularis, SFG
Colak et al. (2019) [43]		13 (13)	16.0 (1.2)	NA	Pure	Medication-free	13 (13)	16.5 (1.3)	NA	Freesurfer	*p* < 0.05 (corrected)	3.0 T	ADHD < TDC: L caudal MFG, R pre-CG
Lu et al. (2019) [44]		53 (53)	10.4 (2.1)	104.0 (5.1)	Pure	Mediation-naïve	53 (53)	10.9 (2.3)	107.6 (6.6)	Freesurfer	*p* < 0.05 (corrected)	3.0 T	-
Vetter et al. (2020) [45]	Pure ADHD	36 (36)	13.0 (1.6)	107.0 (8.0)	Pure	Medication-free	30 (30)	13.6 (1.6)	110.0 (8.0)	Freesurfer	*p* < 0.05 (corrected)	3.0 T	-
	ADHD + ODD/CD	26 (26)	13.0 (1.2)	106.0 (13.0)	ODD, CD	Medication-free	30 (30)	13.6 (1.6)	110.0 (8.0)	Freesurfer	*p* < 0.05 (corrected)	3.0 T	ADHD > TDC: R rostral MFG
Lee et al. (2021) [46]		19 (14)	13.5 (0.9)	113.8 (11.5)	Pure	Medicated (100%)	20 (11)	13.4 (1.2)	110.7 (15.2)	Freesurfer	*p* < 0.05 (corrected)	3.0 T	-
Sarabin et al. (2023) [47]		61 (36)	9.9 (0.7)	NA	behavioral disorder, anxiety disorder	NA	61 (38)	10.0 (0.7)	NA	Freesurfer	*p* < 0.05 (corrected)	3.0 T	-

*ASD* Autistic spectrum disorder, *ADHD* Attention-deficit and hyperactivity disorder, *TDC* Typically developing controls, *IQ* Intelligence quotient, *SD* Standard deviation, *NA* Not available, *T* Tesla, *CIVET* Contentious incident variable entry template, *SDSU* San Diego State University, *NYU* New York University, *SI* Staten Island, *RU* Rutgers University Brain Imaging Centre, *ODD* Oppositional defiant disorder, *CD* Conduct disorder, *L* Left, *R* Right, *SFG* Superior frontal gyrus, *MFG* Middle frontal gyrus, *IFG* Inferior frontal gyrus, *IPL* Inferior parietal lobule, *pre-CG* Precentral gyrus, *OFG* Orbitofrontal gyrus, *SPL* Superior parietal lobule, *para-CG* Paracentral gyrus, *CLASP* Constrained Laplacian Anatomical Segmentation using Proximities

^a^ “Medication-free” represents patients in the study who were taken off medication prior to scanning. “Medication-naive” represents patients who had never been pharmacologically treated

^b^ “CLASP” is the core algorithm for generating the surfaces of gray and white matter in “CIVET” pipeline

### Meta-analysis

Meta-analysis was performed using seed-based d mapping (SDM) software (version 5.15), a meta-analytic tool that has been widely employed in neuroimaging research of various modalities. The procedures of the SDM method have been described in detail elsewhere [48] and its key aspects are described here. First, meta-analysis was separately conducted in ASD and ADHD groups to identify abnormal regional CTh changes relative to healthy individuals in each disorder. These peak coordinates of results reported in Talairach space were first converted to MNI space by SDM software. After that, the SDM software uses the peak coordinates and effect sizes of clusters showing significant differences between patients and controls, including null effect size findings, to create an effect-size signed map and its variance map for each study, represented as an anisotropic Gaussian kernel. Both positive and negative results (increased/decreased CTh in patients than TDC) were reconstructed in the same map to avoid any voxel erroneously appearing positive and negative simultaneously. Then random-effects analysis was performed to obtain the mean map across studies, weighted by sample size, the variance of each study, and estimated between-study heterogeneity. Considering the heterogeneities of clinical characteristics, subgroup analyses were performed based on medication status and comorbidity in each disorder using the same threshold as the pooled meta-analysis, when the subgroups included five or more datasets (*n* ≥ 5) as suggested [49].

A quantitative comparison of CTh was then performed between the two disorders, and standard randomization tests were used to establish statistical significance with mean age and proportion of males as covariances. The conjunction and disjunction analyses were performed to identify overlapping and divergent abnormalities across ASD and ADHD relative to TDC. A random-effects general linear meta-regression was conducted between significant CTh clusters and mean age, mean IQ, and proportion of male patients in each disorder. We also examined linear and nonlinear age-related changes in CTh, as there are non-linear patterns of age-related changes in CTh [50]. Full details of jackknife, heterogeneity and publication bias analysis, and meta-regression analysis are provided in Additional file 1: Supplementary Methods.

All meta-analyses were conducted with the default SDM threshold (*P* < 0.005, *Z* > 1.0 with cluster extent > 10 voxels), which has been found to optimally balance sensitivity and specificity and provide an approximate equivalent to corrected *P* value = 0.05 in SDM [48]. A more stringent probability threshold was employed for meta-regression (*P* < 0.0005) and conjunction and disjunction analyses (*P* < 0.0025) as suggested [48].

## Results

### Study characteristics

**Fig. 1:**
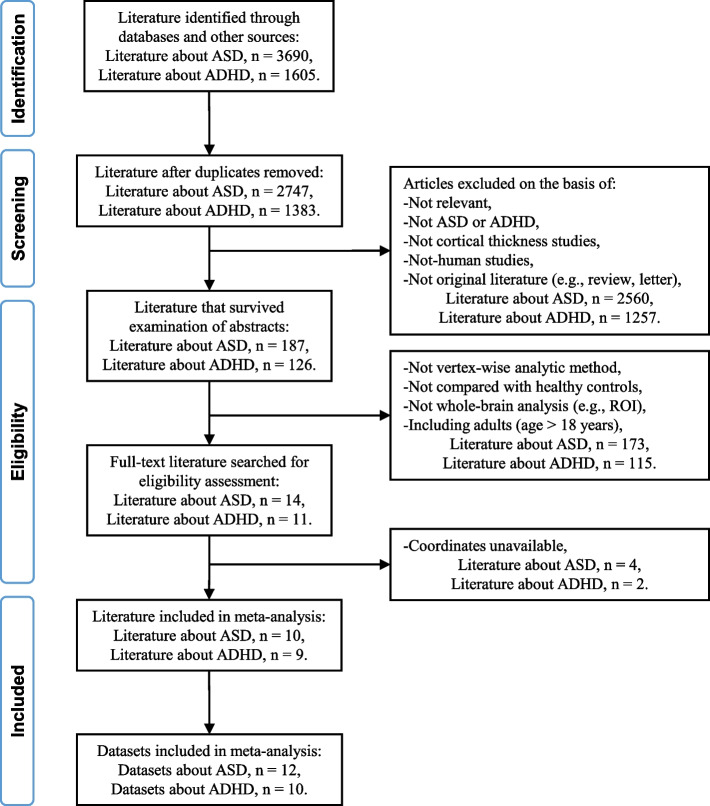
Flowchart of literature search and eligibility assessment

Our search strategy yielded ten ASD studies [9, 30–38] and nine ADHD studies [39–47] that met the abovementioned inclusion criteria (Fig. 1). Among them, two ASD studies [37, 38] and one ADHD study [45] contained two independent patient sample sets. Of note, one ASD study examined both children and adults [41], and we only used the children subgroup dataset in our analyses.

Therefore, a total of 12 ASD datasets involving 458 patients with ASD (age 10.2 ± 3.3 years, IQ 104.1 ± 5.8, males/females 413/45) and 472 controls (age 11.1 ± 2.7 years, IQ 109.8 ± 5.0, males/females 412/60), and 10 ADHD datasets involving 383 patients with ADHD (age 11.4 ± 1.5 years, IQ 103.7 ± 4.4, males/females 326/57) and 380 controls (age 11.5 ± 1.7, IQ 108.0 ± 1.9, males/females 292/88) were included. None of these studies had significant differences in age and sex between patients and controls. In the present study, youth in the two groups with ASD and ADHD had no significant statistical differences in age (*P* > 0.05). The proportion of males in the ASD group is slightly higher than that in the ADHD group (*χ*^2^ = 5.005, *P* = 0.025). Eight datasets did not report if IQ was matched between groups [30, 34, 38, 39, 41, 43, 47], six datasets found significantly higher IQ in controls than patients [9, 31, 32, 37, 40, 42], and there were no between-group differences in IQ in the remaining studies.

As for the medication and comorbidities, there was one ASD dataset recruiting patients who discontinued medications before MR scans [31], and the other ASD studies did not clarify treatment status of their participants. There were two ASD studies included pure ASD patients without any other psychiatric disorders [9, 36], one ASD study reported comorbidities of ADHD and anxiety disorder [38], and the others did not report comorbidity or only excluded patients with neurological or genetic diseases, e.g., tuberous sclerosis and fragile X. In ADHD, there were six ADHD datasets that recruited medication-naïve patients [44] or patients who discontinued medications before MR scans [39, 40, 43, 45]. Three ADHD datasets [41, 42, 46] used medicated patients and one dataset did not clearly indicate the medication status [47]. With regard to comorbidities, six ADHD datasets recruited pure ADHD patients [39, 41, 43–46] while four ADHD datasets reported that some patients had comorbid conduct disorder, oppositional defiant disorder [40, 45], epilepsy [42], and behavioral and anxiety related disorder [47]. It should be noted that patients with a history of cannabis use [43] were not considered as a comorbidity in the current study.

### Meta-analysis

**Fig. 2:**
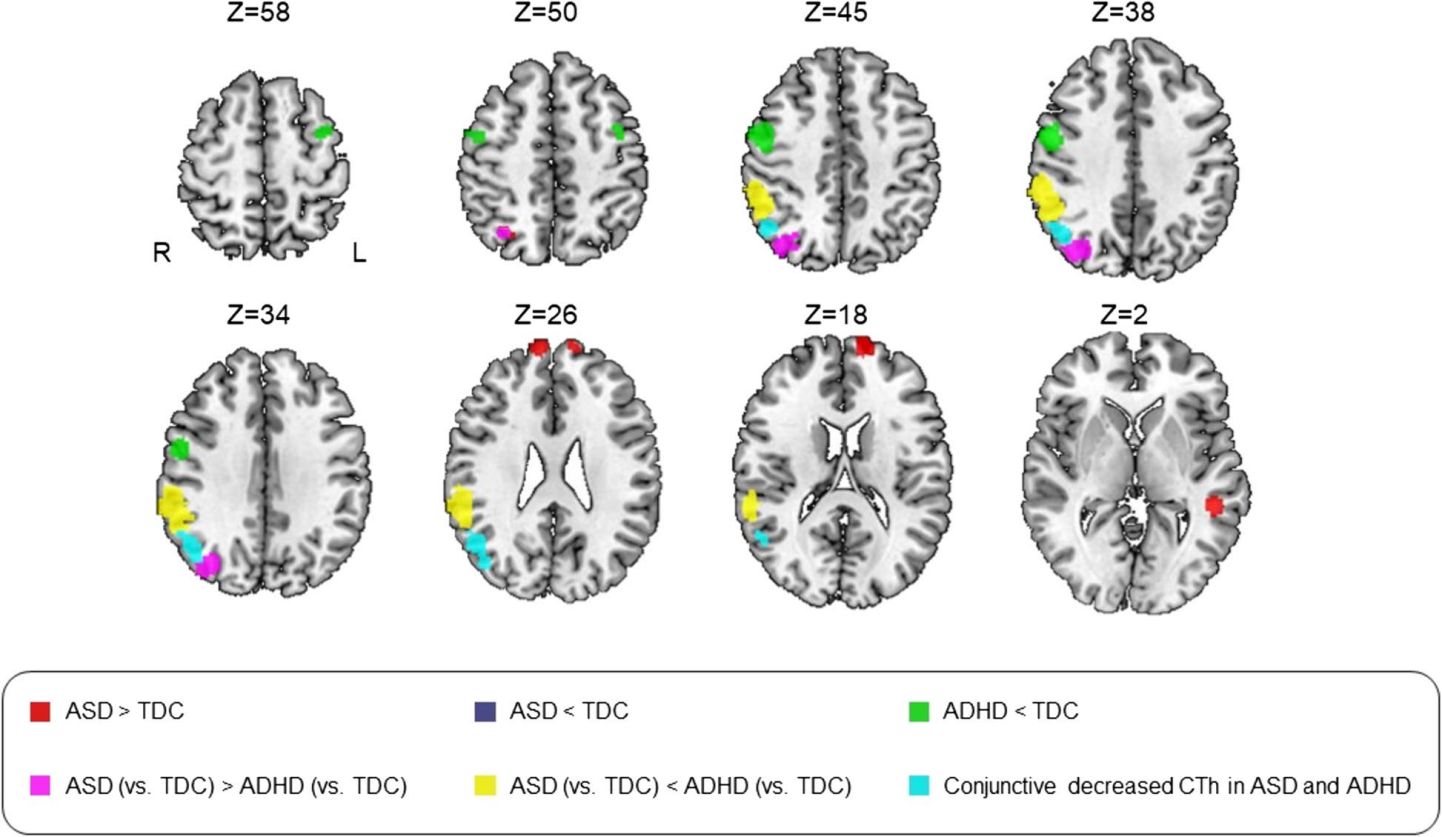
Cortical thickness alterations in ASD and ADHD. The SDM software uses the peak coordinates and effect sizes of clusters showing significant difference between patients and controls to create an effect-size signed map and its variance map for each study. Then random-effects analysis was performed to obtain the mean map of included studies, weighted by sample size, the variance of each study, and between-study heterogeneity. The boundary of the result clusters in the mean map was determined by corresponding statistical thresholds. Subsequently, the results maps of ASD vs controls, ADHD vs controls, and the comparison between ASD and ADHD were mapped onto the Colin 27 brain template to generate Fig. 2

**Fig. 3:**
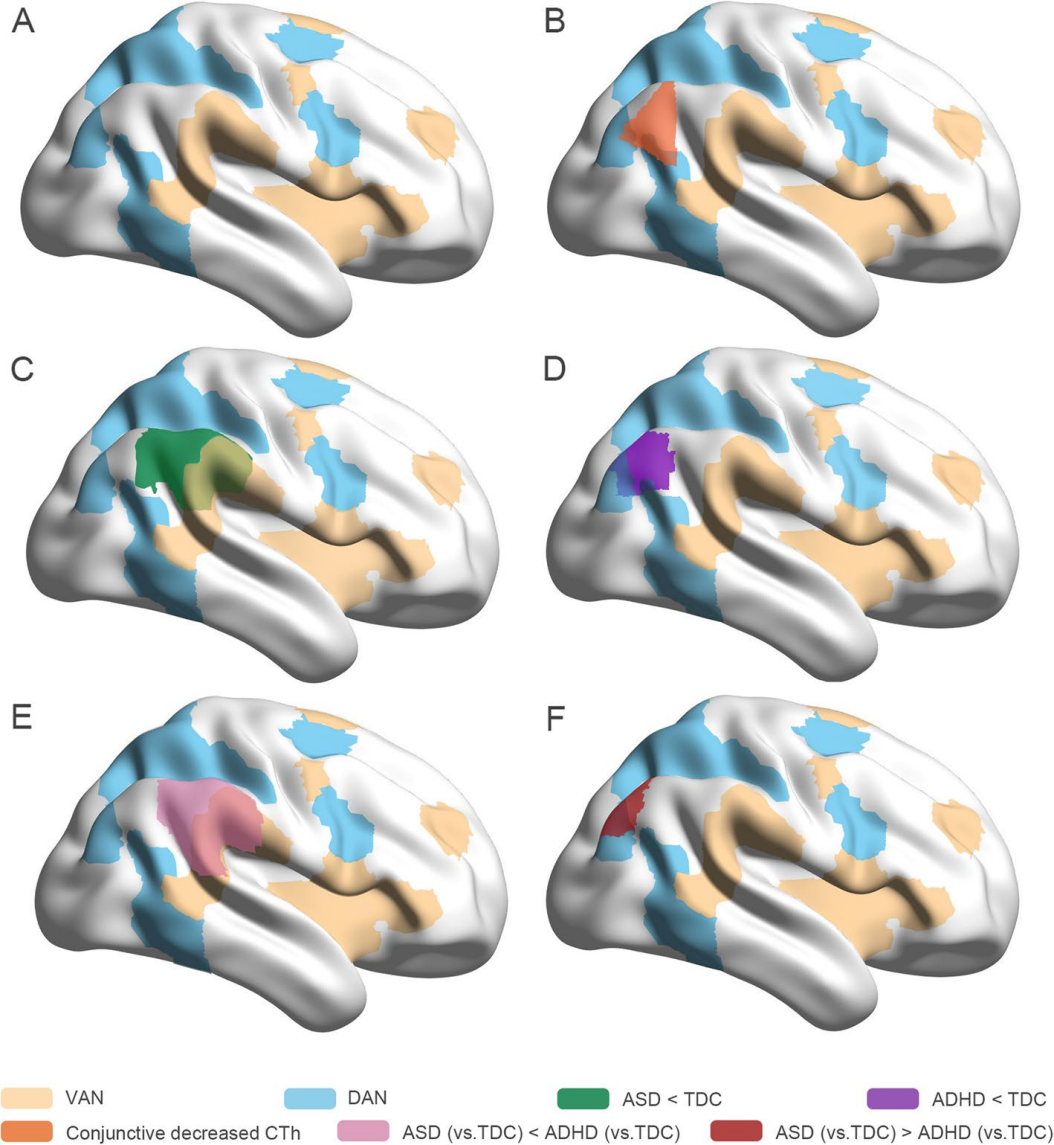
Illustration of CTh alterations in right TPJ in ASD and ADHD in present meta-analysis. The results maps of ASD group, ADHD group, and the comparison and conjunction between ASD and ADHD in right temporoparietal junction (TPJ) were mapped onto the Smoothed International Consortium for Brain Mapping 152 to generate Fig. 3. **A** shows the brain regions of ventral (yellow areas) and dorsal (blue areas) attention networks respectively on the right hemisphere based on Yeo 7 network template [51]. **B** delineates the conjunctively decreased CTh (orange areas) in right TPJ shared in both ASD and ADHD. **C** and **D** demonstrate decreased CTh in right TPJ in ASD (green areas) and ADHD (purple areas) than their respective typically developing controls. **E** and **F** present a more severe CTh decreases in right TPJ in ASD than ADHD (pink areas) and in ADHD than ASD (red areas)

Compared with TDC, patients with ASD showed *increased* CTh in bilateral superior frontal gyrus, left middle temporal gyrus, and right superior parietal lobule (SPL), and *decreased* CTh in right TPJ (Table 2**, **Figs. 2 and 3C**, **Additional file 1: Fig. S1). Egger’s test of funnel plot asymmetry was not statistically significant in all brain regions (all *P* > 0.05), failing to identify publication bias in ASD studies. None of the brain regions with altered CTh showed statistically significant heterogeneity between studies except for the increased CTh in left superior frontal gyrus (*Z* = 1.676, *P* < 0.001). The jackknife sensitivity analysis found that all results were preserved in 11 combinations out of 12 datasets. The number of ASD studies was insufficient for subgroup analysis about medication and comorbidities.

**Table 2:** Differences in cortical thickness among non-adult study participants with ASD, ADHD, and TDC

Region	MNI coordinates			SDM-*Z* value	*P* value	No. of voxels
	*x*	*y*	*z*			
ASD vs. TDC						
ASD > TDC						
Right superior frontal gyrus	8	62	26	1.022	< 0.001	102
Left superior frontal gyrus	− 14	64	20	1.032	< 0.001	175
Left middle temporal gyrus	− 48	− 38	2	1.020	< 0.001	114
Right superior parietal lobule	30	− 62	52	1.020	< 0.001	57
ASD < TDC						
Right temporoparietal junction	58	− 44	36	− 1.530	< 0.001	1264
ADHD vs. TDC						
ADHD < TDC						
Right precentral/postcentral gyrus	54	0	38	− 1.265	0.001	444
Left precentral gyrus	− 38	2	52	− 1.211	0.001	159
Right temporoparietal junction	48	− 66	30	− 1.279	< 0.001	262
ASD (vs. TDC) vs. ADHD (vs. TDC)						
ASD (vs. TDC) > ADHD (vs. TDC)						
Right superior parietal lobule/temporoparietal junction	32	− 72	42	1.040	< 0.001	466
ASD (vs. TDC) < ADHD (vs. TDC)						
Right temporoparietal junction	60	− 44	34	− 1.347	< 0.001	1288
Conjunction						
Right temporoparietal junction	52	− 62	30	–	–	542

*ASD* Autistic spectrum disorder, *ADHD* Attention-deficit and hyperactivity disorder, *MNI* Montreal Neurological Institute, *SDM* Seed-based d Mapping, *TDC* Typically developing controls, *NO *Number

It should be noted that most samples were school-age children and adolescents, except one ASD study analyzed a cohort of preschool-aged children of 2 to 5 years old [30]. In jackknife sensitivity analysis, we excluded the dataset [30] using preschool children. After that, the thickness of left superior frontal gyrus was not significantly increased in ASD compared with TDC. However, in other combinations for sensitivity analysis, the brain regions with increased CTh and their locations remained exactly the same as in the pooled meta-analysis of ASD studies. In linear and nonlinear meta-regression with pooled studies, ASD studies with younger patients related to thicker CTh in left superior frontal gyrus (both regression *P* < 0.0005). After removing the preschool children study [30], the age trend of this brain region was no longer significant, which means the regression result with pooled studies was unstable. No similar relationship was found between CTh and male percentage or mean IQ in this region. Therefore, the increased CTh in left superior frontal gyrus having heterogeneity might come from the influence of the inclusion of preschool children.

Compared with TDC, patients with ADHD showed *decreased* CTh in right precentral gyrus (extending to right postcentral gyrus), left precentral gyrus, and right TPJ (Table 2, Figs. 2 and 3D, Additional file 1: Fig. S2). There was no evidence of publication bias in any cluster. None of the regions with altered CTh showed significant heterogeneity between studies. The jackknife analysis found that decreased CTh in bilateral precentral gyri was preserved in 8 combinations out of 10 datasets. Decreased CTh in the right TPJ remained significant in 9 combinations of ADHD studies. Consistent with the pooled results, the subgroup analysis of patients without comorbidity revealed decreased CTh in right precentral gyrus (extending to right postcentral gyrus), superior frontal gyrus, left precentral gyrus, cingulated gyrus, and bilateral TPJ compared to corresponding TDC (Additional file 1: Table S5). The subgroup analysis of medication-free patients (including medication-naïve patients) revealed no differences in CTh between ADHD and TDC. Linear and nonlinear models of age effects, and effects of mean IQ and percentage of male patients, were all not significantly associated with abnormal CTh in ADHD.

Paralleling the above findings, conjunction analyses revealed a consistent CTh reduction in right TPJ in both ASD and ADHD compared with TDC (Table 2, Figs. 2 and 3B). To follow up this finding, we examined the effects in the different functional subareas of TPJ based on the Yeo 7 network template [51]. From anterior to posterior generally, subareas of TPJ belong to ventral attention network (VAN), default mode network (DMN), and dorsal attention network (DAN) (Fig. 3A). In ASD, CTh was decreased in the right TPJ affiliated with the VAN and DMN, while ADHD CTh reductions in right TPJ were affiliated with the DAN and DMN.

Quantitative comparison between the two disorders revealed that participants with ASD demonstrated thinner CTh in right TPJ in subregions affiliated with the VAN (Fig. 3E) and thicker CTh in right TPJ in subregions affiliated with the DAN and right SPL compared with ADHD (Table 2, Figs. 2 and 3F). No brain regions showed significant disjunctive CTh changes in ASD and ADHD. Furthermore, since there was heterogeneity in the meta-analysis of ASD studies, we discarded the study with preschool children and repeated the comparison of ASD and ADHD to test replicability and reliability of the results. Individuals with ASD also demonstrated more pronounced reductions of CTh in right TPJ in subregions affiliated with the VAN (*P* < 0.001, cluster size = 1337 voxels).

## Discussion

The present meta-analysis identified decreased CTh in a DMN-related subarea of right TPJ that was shared in ASD and ADHD. Effects in other regions differed between the disorders. Direct comparisons of the two disorders revealed that the ASD samples demonstrated increased CTh in right SPL and decreased CTh in the VAN subarea of right TPJ, while the ADHD samples showed reduced CTh in the DAN subarea of right TPJ. These results demonstrate that these two neurodevelopmental disorders have overlapping decreases in CTh in the DMN-affiliated subarea of right TPJ and distinct patterns of CTh abnormalities which represent a basis for understanding the greater problems of perception and social cognition in ASD and the greater behavioral control problems in ADHD. The general pattern of increases in CTh in ASD and decreases of CTh in ADHD also differentiated the disorders.

### Common and distinct features of CTh reduction of right TPJ in ASD and ADHD

The right TPJ is a higher-order area of association cortex including the unimodal visual area V5 responsible for motion processing. TPJ subregions are functionally and anatomically connected with different brain networks [52]. The TPJ region is known to play key roles in integrating polysensory information, biological and general visual motion processing, and it is robustly modulated by top-down attentional control [52]. The activity of right TPJ in DMN has been linked to performance of theory of mind (ToM) tasks [53] and coactivation of medial prefrontal cortex and TPJ in DMN is increased during social cognition [54]. Our findings in TPJ are consistent with the problems of social cognition in both ASD and ADHD [55, 56]. Delayed brain development in right TPJ has been described in both disorders as well, and they have been associated with abnormalities of mentalizing and social abilities in these neurodevelopmental disorders [57, 58]. Thus, the dysmaturation of the TPJ region may be a robust transdiagnostic neuroimaging phenotypic biomarker relevant to the behavioral manifestation of both disorders, albeit in somewhat different ways given the subregions affected.

Impairments of right TPJ in ASD and ADHD have been reported using other neuroimaging modalities. The fractional anisotropy values of white matter between right TPJ and left frontal lobe were reduced in individuals with high-functioning autism and associated with decreased social emotionality [59]. TPJ alterations in ASD have been identified in a magnetoencephalography study which observed impaired connectivity between TPJ and frontal and temporal brain regions during a false-belief task (that is dependent on mentalizing and visual processing) in adults with ASD [60]. Functional MRI (fMRI) studies have identified atypical TPJ responses during visual motion processing [61]. Similarly, in adolescents with ADHD, impaired social cognition and communication have been related to altered functional connectivity between TPJ and precuneus [62].

However, while the TPJ was altered in both disorders, subregion analysis revealed a shared impact of the area associated with the DMN. ASD and ADHD exhibited different alterations in other TPJ subregions that are known to be affiliated with different attention networks [63]. Specifically, in ASD, a separate TPJ region with decreased CTh linked to the VAN was observed, while the additional TPJ reduction in ADHD was located in DAN. This difference implicates different attention network impairments in these two disorders. The VAN mediates the bottom-up attentional processing of novel external stimuli and is involved in detecting and reorienting attention to unexpected stimuli [64]. In contrast, DAN mediates top-down attentional processing involving internal guidance of attention based on prior knowledge, willful plans, and current goals [65]. These anatomic alterations are consistent with psychological studies demonstrating impaired attentional orienting to external stimulation in ASD [66] and difficulties in guiding voluntary allocation of attention in ADHD [67].

### Increased CTh in ASD

Our study found that patients with ASD showed increased CTh in bilateral superior frontal gyrus, left middle temporal gyrus, and right SPL compared with TDC. This suggests a pattern of brain overgrowth or reduced age-related neuronal pruning, in widespread areas of association cortex. Widespread functional alterations of association cortex in ASD have also been reported, though their relation to increases of CTh remains to be fully examined [68, 69]. The longitudinal study has clarified age-related abnormal trajectories of frontal, temporal, and superior parietal CTh in ASD, supporting models of both accelerated thickening and decelerated thinning particularly in early childhood resulting in increased CTh in later life in ASD [6]. Consistent with the neurodevelopmental interpretation of these findings, a neuroanatomical abnormality of a wide range of brain regions has been associated with polygenic risk for ASD [70]. Histological research has indicated that increased CTh in ASD could reflect an excess number of neurons [71] due to reduced synaptic pruning [72]. This neurodevelopmental mechanism might explain the increased CTh observed in the present study and the functional changes of neocortex in older children and adolescents with ASD [15].

Superior frontal abnormalities have been theorized to underlie socialization and cognitive control deficits in ASD [73–75]. Highlighting the divergences between regional increases of the cortical mantle in ASD and decreases in ADHD, the ENIGMA mega-analysis and other studies reported thicker frontal regions were specific to ASD relative to ADHD [73], an effect that has been related to the severity of the autism phenotype [74, 75]. The comparative fMRI meta-analyses of cognitive control between ASD and ADHD have found specific underactivated dorsomedial prefrontal gyrus in ASD [15].

The larger GMV in the left middle temporal gyrus has also been correlated with social and communication deficits [76]. Our findings of increased left middle temporal CTh could partly explain the increased GMV of middle temporal gyrus in ASD observed in previous meta-analyses [77]. The left middle temporal gyrus is involved in language processing. The failure to develop normal language is one of the most common core features of ASD and is correlated with social and communication deficits.

The increase in SPL CTh was specific to ASD. As a region belonging to DAN, this region subserves visual attention and perceptual processes [78]. Taken together with the aberrant CTh in TPJ, its disturbance could account for dysfunctional top-down control of visuospatial attention in ASD [61, 79]. Dysfunctional top-down control of visuospatial attention has been shown to be related to more severe repetitive behavior and restricted interest symptoms [80, 81], and abnormal SPL structural and functional connectivity was one of the most informative features contributing to ASD classification and prediction models [82].

### Decreased CTh in ADHD

In our study, participants with ADHD exhibited reduced CTh in bilateral motor cortices compared with TDC, an effect not observed in ASD. A multicenter research also approved that increased CTh in patients with ASD and thinner cortex in ADHD [21]. Longitudinal studies have shown that the ordered sequence of regional brain development in ADHD is similar to that seen in TDC, but the development was delayed [7, 83]. This is consistent with the clinical observation that many individuals have a reduction in ADHD symptoms by early adulthood, by which time delayed maturation of brain systems may be complete [84]. Similar neurodevelopmental delay has been found in unaffected siblings of children with ADHD, suggesting a hereditary contribution to delayed brain maturation in ADHD [85].

Motor cortices use sensory information to generate adaptive behavioral plans [86] and have been linked to hyperactivity and impulsivity in ADHD [87, 88]. Motor planning, both in its precision and implementation, is altered in ADHD and can contribute to developmental delay of higher-order motor control and impulsivity in ADHD. Correlations between abnormalities in motor cortices and worse performance in motor and response control tasks have been reported previously [89]. The cortical inhibition deficits linked to an alteration in the GABA (γ-aminobutyric acid)-ergic activity in motor cortices in children with ADHD has been reported as a potential mechanism for the observed anatomic alterations in precentral gyrus [90]. Decreased cortical thickness may be able to be alleviated following treatment with psychostimulants, suggesting that ongoing neurochemical and neurophysiological alterations may contribute to this abnormality in ADHD [91].

### Clinical and methodological considerations

Previous GMV meta-analyses reported increased frontal lobe volume and decreased volume in temporal lobe and TPJ in individuals with ASD [77] and widespread decreases in GMV with no regions of increases in individuals with ADHD [15, 92]. Because GMV is more closely associated with cortical surface area, and CTh is relatively stable and distinct from GMV heritably, the current CTh analysis represents an important extension of prior GMV meta-analyses. In the current CTh study, similar patterns of cortical differences between the two neurodevelopmental disorders were also observed. For example, the observed reduction in thickness of the left precentral gyrus in ADHD might partially account for the previously reported volume reduction in the corresponding region [15]. However, previous GMV meta-analyses, which employed young adults, did not reveal overlapping effects in the two disorders, which might be due to the differences in sample age [15, 77, 92]. Indeed, prior research indicated overlap in cortical abnormalities compared to controls existed in children with ASD and ADHD, but not in adult patients [73]. Together, these findings highlight the need for future prospective longitudinal studies employing different cortical metrics to provide a more comprehensive understanding of structural alterations over the course of neurodevelopment.

In addition to identifying differential neurobiological features in ASD and ADHD, the observed altered CTh patterns may have diagnostic implications [93, 94]. For example, with machine learning applications, brain regions related to social and language were considered to be core features in identifying ASD, whereas regions related to motion are core features of ADHD [93, 95]. Additionally, GMV in TPJ was found to classify good and poor responders to methylphenidate treatment in ADHD [96]. We speculate that integrating specific deficits in attention networks associated with TPJ observed in the present study may also be used to increase diagnostic accuracy and improve treatment outcomes in these disorders and warrants further exploration.

It should be noted that in this study, we were unable to exclude the possibility that the effects observed in patients were influenced by sex bias. In typically developing populations, sex has significant influences on the development of several brain regions, including prefrontal cortex and TPJ [97]. For example, females have thicker CTh in TPJ than age-matched males from late childhood and consistently through old ages [98, 99]. It is common to include more males in ASD and ADHD studies due to the higher prevalence of both disorders in males. Sex has been demonstrated to impact the neuroanatomical alterations in ASD [100] and ADHD [29] both in effect extent and location and shape brain morphology during development. For example, males with ASD were characterized by cortical thickening while females exhibit cortical thinning [101]. Males with ASD have more significant temporal lobe gray matter enlargement compared with females [102], suggesting ASD males have more severe social and communication defects [100]. In ADHD, males have poorer motor performance than females which is related to a smaller premotor surface area in males [103]. However, the study design of the included original studies precluded us from performing subgroup analyses in male and female patients respectively. Larger respective studies of males and females are needed to better explore the potential impact of sex on the CTh alterations observed in these conditions [104].

Although ASD and ADHD are dynamic disorders with complex cortical changes over time from childhood into adulthood [7, 50, 70], we did not observe a significant association between age and altered CTh in the meta-regression analysis. This might be because only average age in study samples was extracted from each study for these analyses, which has limited the ability to precisely characterize age effects on brain CTh in ASD and ADHD. Nevertheless, we acknowledge that age is a crucial factor for brain developmental trajectories in neurodevelopmental disorders, and the greater heterogeneity found in preschool children in our analysis supports age-related effects.

Regarding methodological consideration, eligible studies included in our meta-analysis used two mainstream preprocessing methods, Freesurfer and CIVET. The geometrical accuracy of surface extraction is critical for the accurate measurement of CTh which could result in undetectable potential method heterogeneity. A comparative study found that CIVET reconstructs the most accurate surfaces and Freesurfer offers more realistic surfaces [105]. While potential differences exist between the two methods, they both demonstrate good geometric estimation of cortical surfaces. Similarly, the reconstruction of cortices was influenced by field strength and sequence parameters, especially the repetition time, which warrants consideration. These factors might subtly affect the contrast between gray and white matter and the extraction of white matter surface and the pial surfaces. While considered and evaluated statistically (i.e., significant heterogeneity in ASD), these methodological differences across studies represent an important consideration when interpreting our findings.

This meta-analysis has other limitations. First, the representativeness of the meta-analysis may be limited by the fact that many studies recruited high-functioning ASD individuals in neuroimaging research to promote successful MRI studies. This limits the ability to generalize the reported neuroimaging results to ASD with more severe behavioral and intellectual disabilities. Second, the effects of medication exposure cannot be explored by meta-regression and subgroup analysis in ASD groups because all studies did not report precise type and dose of medication, and only one study in ASD reported medicated status. Although we examined medication and comorbidity effects in ADHD studies, the statistical power was limited by the number of studies. Differences in psychotropic medication exposure between ASD and ADHD groups may have contributed to the differences between the two disorders. Third, although we did not find statistical differences in mean age between ASD and ADHD, the conjunctive results should be treated conservatively when considering the subtle mean age difference between ASD and ADHD groups. Fourth, the results of the original studies were reported in the standard space of the mature brain, and the use of a mask specifically created for children and adolescents would more accurately estimate spatial changes in the developing brain. Fifth, the current disorder-compared results are preliminary and indirect due to the scarcity of original studies comparing the two disorders in the same study. While our exploration could guide the design and further investigations of transdiagnostic studies. Last, more neuroimaging studies linking structural and functional alterations by using multimodal brain MRI methods [106–111] to better understand the functional effects of observed anatomic alterations are needed.

## Conclusions

The case–control meta-analyses of ASD and ADHD found shared decreases in CTh in a subarea of right TPJ affiliated with the DMN. Other subregions of the TPJ were differentially affected in ASD and ADHD, which may explain divergent disturbances of attention in the two disorders. Other neocortical alterations in ADHD involved a thinning of CTh in motor cortices, while alterations of ASD involved increases of CTh in association cortices, highlighting a dramatic differentiation of neuroanatomic alterations in these two neurodevelopmental disorders. Our findings contribute to the understanding of differential and overlapping alterations of brain maturation in ASD and ADHD, which is important for the elucidation of disorder-specific etiologies.

### Supplementary Information

**Additional file 1.** Supplementary methods of sensitivity, heterogeneity, publication bias, and meta-regression analyses. **Table S1.** PRISMA 2020 Checklist. **Table S2.** List of excluded studies that meet other inclusion criteria. **Table S3.** The checklist of imaging methodology quality assessment for all the articles included in the present meta-analysis. **Table S4.** Data sources of all public database studies included in the present meta-analysis. **Table S5.** Differences in cortical thickness between pure ADHD without comorbidity and TDC. **Fig. S1.** Results of cortical thickness differences between ASD and TDC. **Fig. S2.** Results of cortical thickness differences between ADHD and TDC.

## Data Availability

This study generated and analyzed the summary statistics of previous published studies. All data are available in these included articles. The statistical data of meta-analysis that support the findings of this study are available from the corresponding authors upon reasonable request.

## Abbreviations

ADHD: Attention-deficit/hyperactivity disorder
ASD: Autism spectrum disorder
CTh: Cortical thickness
DAN: Dorsal attention network
DMN: Default mode network
fMRI: Functional MRI
GMV: Gray matter volume
MNI: Montreal Neurological Institute
MRI: Magnetic resonance imaging
PRISMA: Preferred Reporting Items for Systematic Reviews and Meta-Analyses
PROSPERO: Prospective register of systematic reviews
SDM: Seed-based d mapping
SPL: Superior parietal lobule
TDC: Typically developing controls
TPJ: Temporoparietal junction
VAN: Ventral attention network
