# Supplementary material for: Common and distinct cortical thickness alterations in youth with autism spectrum disorder and attention-deficit/hyperactivity disorder
Source: BMC Med. 2024 Mar 4;22:92. doi: 10.1186/s12916-024-03313-2 (PMC10910790; doi:10.1186/s12916-024-03313-2)

**Additional file 1**

[**Supplementary Methods** 2](#_Toc159426839)

[**Sensitivity, heterogeneity, and publication bias analyses** 2](#_Toc159426840)

[**Meta-regression analysis** 2](#_Toc159426841)

[**Table S1.** PRISMA 2020 Checklist* 3](#_Toc159426842)

[**Table S2.** List of excluded studies that meet other inclusion criteria. 7](#_Toc159426843)

[**Table S3.** The checklist of imaging methodology quality assessment for all the articles included in the present meta-analysis. 9](#_Toc159426844)

[**Table S4.** Data sources of all public database studies included in the present meta-analysis. 11](#_Toc159426845)

[**Table S5.** Differences in cortical thickness between pure ADHD without comorbidity and TDC. 12](#_Toc159426846)

[**Figure S1.** Results of cortical thickness differences between ASD and TDC. 13](#_Toc159426847)

[**Figure S2.** Results of cortical thickness differences between ADHD and TDC. 14](#_Toc159426848)

**Supplementary Methods**

**Sensitivity, heterogeneity, and publication bias analyses**

In each of the meta-analyses of ASD and ADHD, a jackknife sensitivity analysis was conducted to test the robustness of results by iteratively repeating the analyses, discarding one dataset each time using the same threshold as the pooled meta-analysis. If a brain region of meta-analysis remains significant in all or most of the combinations of studies, this finding is considered highly replicable. Also, in each meta-analysis, the between-study heterogeneity of each cluster was examined using a random-effects model with Q statistics and tested with a permutation approach (a voxel threshold of P < 0.005, peak Z > 1, cluster extent > 10 voxels), findings within these regions were considered showing significant heterogeneity. Publication bias was estimated with Egger’s test to assess the asymmetry of the funnel plot of each cluster in patient-control comparison, and any result showing P < 0.05 was defined as significant for publication bias.

**Meta-regression analysis**

The random-effects general linear meta-regression in SDM was conducted between significant clusters and mean age, mean IQ, and percentage of male patients in each disorder to explore their potential effects on CTh alternations. And we also examined the relationship between mean age-squared and altered CTh. The probability threshold was set as P <0.0005 to control false positives. Eligible findings should be detected in both the slope and one of the extreme values of the regression variables, and the ones not found in the pooled meta-analysis will be discarded. Moreover, the regression plots would be visually inspected to exclude the findings caused by too few studies.

**Table S1.** PRISMA 2020 Checklist*

| **Section/topic** | **Item#** | **Checklist item** | **Location where item is reported** |
| --- | --- | --- | --- |
| **TITLE** | | |  |
| Title | 1 | Identify the report as a systematic review. | None |
| **ABSTRACT** | | |  |
| Structured summary | 2 | See the PRISMA 2020 for Abstracts checklist. | Abstract |
| **INTRODUCTION** | | |  |
| Rationale | 3 | Describe the rationale for the review in the context of existing knowledge. | Introduction Paragraph (P) 1-4 |
| Objectives | 4 | Provide an explicit statement of the objective(s) or question(s) the review addresses. | Introduction P5 |
| **METHODS** | | |  |
| Eligibility criteria | 5 | Specify the inclusion and exclusion criteria for the review and how studies were grouped for the syntheses. | Method: Search strategy and study inclusion |
| Information sources | 6 | Specify all databases, registers, websites, organisations, reference lists and other sources searched or consulted to identify studies. Specify the date when each source was last searched or consulted. | Method: Search strategy and study inclusion |
| Search strategy | 7 | Present the full search strategies for all databases, registers and websites, including any filters and limits used. | Method: Search strategy and study inclusion |
| Selection process | 8 | Specify the methods used to decide whether a study met the inclusion criteria of the review, including how many reviewers screened each record and each report retrieved, whether they worked independently, and if applicable, details of automation tools used in the process. | Method: Search strategy and study inclusion |
| Data collection process | 9 | Specify the methods used to collect data from reports, including how many reviewers collected data from each report, whether they worked independently, any processes for obtaining or confirming data from study investigators, and if applicable, details of automation tools used in the process. | Method: Search strategy and study inclusion |
| Data items | 10a | List and define all variables for which data were sought (e.g., PICOS, funding sources) and any assumptions and simplifications made. | Method: Search strategy and study inclusion; Table 1 |
|  | 10b | List and define all other variables for which data were sought (e.g. participant and intervention characteristics, funding sources). Describe any assumptions made about any missing or unclear information. | Supplementary Results: Included studies and sample characteristics |
| Study risk of bias assessment | 11 | Specify the methods used to assess risk of bias in the included studies, including details of the tool(s) used, how many reviewers assessed each study and whether they worked independently, and if applicable, details of automation tools used in the process. | Supplementary Methods: Sensitivity, heterogeneity, and publication bias analyses |
| Effect measures | 12 | Specify for each outcome the effect measure(s) (e.g. risk ratio, mean difference) used in the synthesis or presentation of results. | Method: Meta-analysis |
| Synthesis methods | 13a | Describe the processes used to decide which studies were eligible for each synthesis (e.g. tabulating the study intervention characteristics and comparing against the planned groups for each synthesis (item #5)). | Method: Search strategy and study inclusion |
|  | 13b | Describe any methods required to prepare the data for presentation or synthesis, such as handling of missing summary statistics, or data conversions. | Method: SDM Meta-analysis |
|  | 13c | Describe any methods used to tabulate or visually display results of individual studies and syntheses. | None |
|  | 13d | Describe any methods used to synthesize results and provide a rationale for the choice(s). If meta-analysis was performed, describe the model(s), method(s) to identify the presence and extent of statistical heterogeneity, and software package(s) used. | Method: SDM Meta-analysis; Supplementary Methods: Sensitivity, heterogeneity, and publication bias analyses |
|  | 13e | Describe any methods used to explore possible causes of heterogeneity among study results (e.g. subgroup analysis, meta-regression). | Supplementary Methods: Sensitivity, heterogeneity, and publication bias analyses; Supplementary Results: Exploration of regions showing significant heterogeneity |
|  | 13f | Describe any sensitivity analyses conducted to assess robustness of the synthesized results. | Supplementary Methods: Sensitivity, heterogeneity, and publication bias analyses |
| Reporting bias assessment | 14 | Describe any methods used to assess risk of bias due to missing results in a synthesis (arising from reporting biases). | Supplementary Methods: Sensitivity, heterogeneity, and publication bias analyses |
| Certainty assessment | 15 | Describe any methods used to assess certainty (or confidence) in the body of evidence for an outcome. | None |
| **RESULTS** | | |  |
| Study selection | 16a | Describe the results of the search and selection process, from the number of records identified in the search to the number of studies included in the review, ideally using a flow diagram. | Results: Study characteristics; Figure 1 |
|  | 16b | Cite studies that might appear to meet the inclusion criteria, but which were excluded, and explain why they were excluded. | Supplementary Methods: Quality assessment; Table S3 |
| Study characteristics | 17 | Cite each included study and present its characteristics. | Results: Study characteristics; Table 1 |
| Risk of bias in studies | 18 | Present assessments of risk of bias for each included study. | Results: Meta-analysis |
| Results of individual studies | 19 | For all outcomes, present, for each study: (a) summary statistics for each group (where appropriate) and (b) an effect estimate and its precision (e.g. confidence/credible interval), ideally using structured tables or plots. | Table 2 |
| Results of syntheses | 20a | For each synthesis, briefly summarise the characteristics and risk of bias among contributing studies. | Results: Study characteristics, Limitations; Supplementary Results: Included studies and sample characteristics |
|  | 20b | Present results of all statistical syntheses conducted. If meta-analysis was done, present for each the summary estimate and its precision (e.g. confidence/credible interval) and measures of statistical heterogeneity. If comparing groups, describe the direction of the effect. | Results: Meta-analysis; Table 2; Figure2; Figure 3 |
|  | 20c | Present results of all investigations of possible causes of heterogeneity among study results. | Supplementary Results: Exploration of regions showing significant heterogeneity |
|  | 20d | Present results of all sensitivity analyses conducted to assess the robustness of the synthesized results. | Results: Meta-analysis |
| Reporting biases | 21 | Present assessments of risk of bias due to missing results (arising from reporting biases) for each synthesis assessed. | Results: Meta-analysis |
| Certainty of evidence | 22 | Present assessments of certainty (or confidence) in the body of evidence for each outcome assessed. | Results: Meta-analysis; Table 2 |
| **DISCUSSION** | | |  |
| Discussion | 23a | Provide a general interpretation of the results in the context of other evidence. | Discussion P1 |
|  | 23b | Discuss any limitations of the evidence included in the review. | Discussion P11 |
|  | 23c | Discuss any limitations of the review processes used. | Discussion P11 |
|  | 23d | Discuss implications of the results for practice, policy, and future research. | Discussion P11-12 |
| **OTHER INFORMATION** | | |  |
| Registration and protocol | 24a | Provide registration information for the review, including register name and registration number, or state that the review was not registered. | Method: Search strategy and study inclusion |
|  | 24b | Indicate where the review protocol can be accessed, or state that a protocol was not prepared. | Method: Search strategy and study inclusion, Availability of Data and Materials |
|  | 24c | Describe and explain any amendments to information provided at registration or in the protocol. | None |
| Support | 25 | Describe sources of financial or non-financial support for the review, and the role of the funders or sponsors in the review. | Acknowledgments |
| Competing interests | 26 | Declare any competing interests of review authors. | Conflict of Interest |
| Availability of data, code and other materials | 27 | Report which of the following are publicly available and where they can be found: template data collection forms; data extracted from included studies; data used for all analyses; analytic code; any other materials used in the review. | Availability of Data and Materials |

*Note: Page MJ, McKenzie JE, Bossuyt PM, Boutron I, Hoffmann TC, Mulrow CD, et al. The PRISMA 2020 statement: an updated guideline for reporting systematic reviews. BMJ 2021;372:n71. doi: 10.1136/bmj.n71

**Table S2.** List of excluded studies that meet other inclusion criteria.

| Group | Reason for exclusion | Study |
| --- | --- | --- |
| **ASD studies** | 1. The age of all or partial participants was over 18. And the studies did not provide coordinates of the cortical thickness (CTh) results. | Wallace GL, Dankner N, Kenworthy L, Giedd JN, Martin A. Age-related temporal and parietal cortical thinning in autism spectrum disorders. Brain : a journal of neurology. 2010;133(12):3745-54. |
|  |  | Ecker C, Ginestet C, Feng Y, Johnston P, Lombardo MV, Lai MC, et al. Brain surface anatomy in adults with autism: the relationship between surface area, cortical thickness, and autistic symptoms. JAMA psychiatry. 2013;70(1):59-70. |
|  |  | Ecker C, Shahidiani A, Feng Y, Daly E, Murphy C, D'Almeida V, et al. The effect of age, diagnosis, and their interaction on vertex-based measures of cortical thickness and surface area in autism spectrum disorder. Journal of Neural Transmission. 2014;121(9):1157-70. |
|  |  | Libero LE, DeRamus TP, Deshpande HD, Kana RK. Surface-based morphometry of the cortical architecture of autism spectrum disorders: Volume, thickness, area, and gyrification. Neuropsychologia. 2014;62(1):1-10. |
|  |  | Zoltowski AR, Lyu I, Failla M, Mash LE, Dunham K, Feldman JI, et al. Cortical Morphology in Autism: Findings from a Cortical Shape-Adaptive Approach to Local Gyrification Indexing. Cerebral cortex (New York, NY : 1991). 2021;31(11):5188-205. |
|  | 1. The age of all or partial participants was over 18. | Scheel C, Rotarska-Jagiela A, Schilbach L, Lehnhardt FG, Krug B, Vogeley K, et al. Imaging derived cortical thickness reduction in high-functioning autism: Key regions and temporal slope. NeuroImage. 2011;58(2):391-400. |
|  |  | Doyle-Thomas KAR, Duerden EG, Taylor MJ, Lerch JP, Soorya LV, Wang AT, et al. Effects of age and symptomatology on cortical thickness in autism spectrum disorders. Research in autism spectrum disorders. 2013;7(1):141-50. |
|  |  | Balardin JB, Sato JR, Vieira G, Feng Y, Daly E, Murphy C, et al. Relationship Between Surface-Based Brain Morphometric Measures and Intelligence in Autism Spectrum Disorders: Influence of History of Language Delay. Autism Research. 2015;8(5):556-66. |
|  |  | Gebauer L, Foster NEV, Vuust P, Hyde KL. Is there a bit of autism in all of us? Autism spectrum traits are related to cortical thickness differences in both autism and typical development. Research in autism spectrum disorders. 2015;13-14:8-14. |
|  |  | Schaer M, Kochalka J, Padmanabhan A, Supekar K, Menon V. Sex differences in cortical volume and gyrification in autism. Molecular Autism. 2015;6 (1) (no pagination)(42). |
|  |  | Valk SL, Di Martino A, Milham MP, Bernhardt BC. Multicenter mapping of structural network alterations in autism. Human brain mapping. 2015;36(6):2364-73. |
|  |  | Koolschijn PCMP, Geurts HM. Gray Matter Characteristics in Mid and Old Aged Adults with ASD. Journal of Autism and Developmental Disorders. 2016;46(8):2666-78. |
|  |  | Pereira AM, Campos BM, Coan AC, Pegoraro LF, de Rezende TJR, Obeso I, et al. Differences in cortical structure and functional MRI connectivity in high functioning autism. Frontiers in Neurology. 2018;9 (JUL) (no pagination)(539). |
|  |  | Maier S, Tebartz van Elst L, Perlov E, Düppers AL, Nickel K, Fangmeier T, et al. Cortical properties of adults with autism spectrum disorder and an IQ>100. Psychiatry Research: Neuroimaging. 2018;279:8-13. |
|  |  | Kohli JS, Kinnear MK, Martindale IA, Carper RA, Muller RA. Regionally decreased gyrification in middle-aged adults with autism spectrum disorders. Neurology. 2019;11. |
|  |  | Ecker C, Pretzsch CM, Bletsch A, Mann C, Schaefer T, Ambrosino S, et al. Interindividual Differences in Cortical Thickness and Their Genomic Underpinnings in Autism Spectrum Disorder. The American journal of psychiatry. 2022 Mar;179(3):242-254. |
|  |  | James D, Lam VT, Jo B, Fung LK. Region-specific associations between gamma-aminobutyric acid A receptor binding and cortical thickness in high-functioning autistic adults. Autism Research. 2022;15(6):1068-82. |
|  |  | Pretzsch CM, Schafer T, Lombardo MV, Warrier V, Mann C, Bletsch A, et al. Neurobiological Correlates of Change in Adaptive Behavior in Autism. The American journal of psychiatry. 2022;179(5):336-49. |
|  |  | Hong S-J, Mottron L, Park B-Y, Benkarim O, Valk SL, Paquola C, et al. A convergent structure-function substrate of cognitive imbalances in autism. Cerebral Cortex. 2023 Feb 20;33(5):1566-1580. |
|  | 1. The studies did not provide coordinates of the CTh results. | Sharda M, Foster NEV, Tryfon A, Doyle-Thomas KAR, Ouimet T, Anagnostou E, et al. Language Ability Predicts Cortical Structure and Covariance in Boys with Autism Spectrum Disorder. Cerebral cortex (New York, NY : 1991). 2017;27(3):1849-62. |
|  |  | Pappaianni E, Siugzdaite R, Vettori S, Venuti P, Job R, Grecucci A. Three shades of grey: detecting brain abnormalities in children with autism using source-, voxel- and surface-based morphometry. European Journal of Neuroscience. 2018;47(6):690-700. |
|  |  | Li D, Liu C, Huang Z, Li H, Xu Q, Zhou B, et al. Common and Distinct Disruptions of Cortical Surface Morphology Between Autism Spectrum Disorder Children With and Without SHANK3 Deficiency. Frontiers in neuroscience. 2021;15:751364. |
|  | 1. The study did not provide whether the effect size is positive of negative. | Chen J, Wei Z, Liang C, Liu B, Guo J, Kong X, et al. Dysfunction of the Auditory Brainstem as a Neurophysiology Subtype of Autism Spectrum Disorder. Frontiers in neuroscience. 2021;15:637079. |
| **ADHD studies** | 1. The age of all participants was over 18. | Proal E, Reiss PT, Klein RG, Mannuzza S, Gotimer K, Ramos-Olazagasti MA, et al. Brain gray matter deficits at 33-year follow-up in adults with attention-deficit/hyperactivity disorder established in childhood. Archives of general psychiatry. 2011;68(11):1122-34. |
|  |  | Lisdahl KM, Tamm L, Epstein JN, Jernigan T, Molina BS, Hinshaw SP, et al. The impact of ADHD persistence, recent cannabis use, and age of regular cannabis use onset on subcortical volume and cortical thickness in young adults. Drug and alcohol dependence. 2016;161:135-46. |
|  |  | Cherkasova MV, Faridi N, Casey KF, Larcher K, O'Driscoll GA, Hechtman L, et al. Differential Associations between Cortical Thickness and Striatal Dopamine in Treatment-Naïve Adults with ADHD vs. Healthy Controls. Frontiers in human neuroscience. 2017;11:421 |
|  | 1. The studies did not provide coordinates of the CTh results | Narr KL, Woods RP, Lin J, Kim J, Phillips OR, Del'Homme M, et al. Widespread cortical thinning is a robust anatomical marker for attention-deficit/hyperactivity disorder. Journal of the American Academy of Child and Adolescent Psychiatry. 2009;48(10):1014-22. |
|  |  | Almeida Montes LG, Prado Alcántara H, Martínez García RB, De La Torre LB, Avila Acosta D, Duarte MG. Brain cortical thickness in ADHD: age, sex, and clinical correlations. Journal of attention disorders. 2013;17(8):641-54. |

**Table S3.** The checklist of imaging methodology quality assessment for all the articles included in the present meta-analysis.

| **12-point checklist** | ASD |  |  |  |  |  |  |  |  |  |  |  |  | ADHD |  |  |  |  |  |  |  |  |  |
| --- | --- | --- | --- | --- | --- | --- | --- | --- | --- | --- | --- | --- | --- | --- | --- | --- | --- | --- | --- | --- | --- | --- | --- |
|  | Raznahan | Duerden | Schaer | Dierker | Foster | Sussman | Yang | Tanigawa | Kohli SDSU | Kohli NYU | Yin SI | Yin RU |  | Qiu | de Zeeuw | Hoekzema children | Saute | Colak | Lu | Vetter Pure | Vetter ODD/CD | Lee | Sarabin |
| **Category 1: Subjects** |  |  |  |  |  |  |  |  |  |  |  |  |  |  |  |  |  |  |  |  |  |  |  |
| 1. Patients were evaluated prospectively, specific diagnostic criteria were applied, and demographic data were reported | 1 | 1 | 1 | 1 | 1 | 0.5 | 1 | 1 | 1 | 1 | 1 | 1 |  | 1 | 1 | 1 | 1 | 1 | 1 | 1 | 1 | 1 | 1 |
| 2. Healthy comparison participants were evaluated prospectively; psychiatric and medical illnesses were excluded | 1 | 1 | 0.5 | 1 | 1 | 1 | 1 | 1 | 1 | 1 | 1 | 1 |  | 1 | 1 | 1 | 1 | 1 | 1 | 1 | 1 | 1 | 1 |
| 3. Important variables (e.g., age, gender, drug status, and symptom severity) were checked either via stratification or statistics | 0.5 | 1 | 0.5 | 0.5 | 0.5 | 0.5 | 0.5 | 0.5 | 0.5 | 0.5 | 0.5 | 0.5 |  | 0.5 | 1 | 1 | 1 | 0.5 | 1 | 1 | 1 | 1 | 0.5 |
| 4. All patients were comorbidity free (all comorbid-free 1, exclude neurology or gene 0.5, comorbid psychiatry or non-report 0) | 0.5 | 0 | 0.5 | 0.5 | 1 | 0.5 | 0.5 | 1 | 0.5 | 0 | 0 | 0 |  | 1 | 0 | 1 | 0 | 1 | 1 | 1 | 0 | 1 | 0 |
| 5. All patients were medication free (all medication-free 1, partial medicated 0.5, all medicated or non-report 0) | 0 | 1 | 0 | 0 | 0 | 0 | 0 | 0 | 0 | 0 | 0 | 0 |  | 1 | 1 | 0.5 | 0.5 | 1 | 1 | 1 | 1 | 0 | 0 |
| 6. Sample size per group: ≥ 20, scores 1; ≥ 10, scores 0.5 | 1 | 1 | 0.5 | 1 | 1 | 1 | 1 | 0.5 | 1 | 1 | 0.5 | 0.5 |  | 0.5 | 1 | 1 | 0.5 | 0.5 | 1 | 1 | 1 | 0.5 | 1 |
| **Category 2: Methods for image acquisition and analysis** | | | |  |  |  |  |  |  |  |  |  |  |  |  |  |  |  |  |  |  |  |  |
| 7. Magnet strength: 3T, scores 1; 1.5T, scores 0.5 | 0.5 | 0.5 | 1 | 1 | 1 | 1 | 1 | 1 | 1 | 1 | 0.5 | 1 |  | 1 | 0.5 | 0.5 | 0.5 | 1 | 1 | 1 | 1 | 1 | 1 |
| 8. The imaging technique used was clearly described so that it could be reproduced | 1 | 1 | 1 | 1 | 0.5 | 1 | 1 | 1 | 1 | 1 | 1 | 1 |  | 1 | 1 | 1 | 1 | 1 | 1 | 0.5 | 0.5 | 1 | 1 |
| 9. Whole brain analysis was automated without a previously defined region | 1 | 1 | 1 | 1 | 1 | 1 | 1 | 1 | 1 | 1 | 1 | 1 |  | 1 | 1 | 1 | 1 | 1 | 1 | 1 | 1 | 1 | 1 |
| 10. Spatial coordinates were reported in a standard space (e.g., Talairach or MNI coordinates) | 1 | 1 | 1 | 1 | 1 | 1 | 1 | 1 | 1 | 1 | 1 | 1 |  | 1 | 1 | 1 | 1 | 1 | 1 | 1 | 1 | 1 | 1 |
| **Category 3: Results and conclusions** |  |  |  |  |  |  |  |  |  |  |  |  |  |  |  |  |  |  |  |  |  |  |  |
| 11. Statistical results were corrected for multiple comparison scores 1, uncorrected scores 0.5 | 1 | 1 | 1 | 1 | 0.5 | 1 | 1 | 1 | 1 | 1 | 1 | 1 |  | 0.5 | 1 | 1 | 1 | 1 | 1 | 1 | 1 | 1 | 1 |
| 12. Conclusions were consistent with the results obtained, and the limitations were discussed | 1 | 0.5 | 1 | 1 | 0.5 | 1 | 1 | 1 | 1 | 1 | 1 | 1 |  | 1 | 0.5 | 1 | 1 | 1 | 1 | 1 | 1 | 1 | 1 |
| **Total score** | 9.5 | 10 | 9 | 10 | 9 | 9.5 | 10 | 10 | 10 | 9.5 | 8.5 | 9 |  | 10.5 | 10 | 11 | 9.5 | 11 | 12 | 11.5 | 10.5 | 10.5 | 9.5 |

**Table S4.** Data sources of all public database studies included in the present meta-analysis.

| Group | Study | Public database |
| --- | --- | --- |
| **ASD studies** | Kohli et al. (2019) Data from NYU | Autism Brain Imaging Data Exchange (ABIDE) from the New York University site |
|  | Yin et al. (2022) Data from SI | Child Mind Institute Healthy Brain Network dataset (HBN) from Staten Island site |
|  | Yin et al. (2022) Data from RU | Child Mind Institute Healthy Brain Network dataset (HBN) from Rutgers University Brain Imaging Centre |
| **ADHD studies** | Sarabin et al. (2023) | Adolescent Brain Cognitive Development (ABCD) Database |

**Table S5.** Differences in cortical thickness between pure ADHD without comorbidity and TDC.

| Region | MNI coordinates | | | SDM-Z value | *P* value | No. of voxels |
| --- | --- | --- | --- | --- | --- | --- |
|  | x | y | z |  |  |  |
| **ADHD < TDC** | | | | | | |
| Right precentral/postcentral gyrus | 56 | -10 | 38 | -1.482 | < 0.001 | 802 |
| Left precentral gyrus | -40 | 6 | 50 | -1.483 | <0.001 | 243 |
| Left cingulated gyrus | -6 | -80 | 12 | -1.229 | 0.002 | 35 |
| Left temporoparietal junction | -46 | -66 | 46 | -1.229 | 0.002 | 35 |
| Right superior frontal gyrus | 28 | 66 | -10 | -1.228 | 0.002 | 35 |
| Left cingulated gyrus | -10 | -90 | 4 | -1.229 | 0.002 | 23 |
| Right temporoparietal junction | 42 | -70 | 24 | -1.229 | 0.002 | 21 |
| Right temporoparietal junction | 28 | -64 | 44 | -1.229 | 0.002 | 13 |

Abbreviations: ADHD, attention deficit and hyperactivity disorder; TDC, typically developing controls; MNI, Montreal Neurological Institute; SDM, Seed-based d Mapping; NO., number.

**Figure S1.** Results of cortical thickness differences between ASD and TDC.


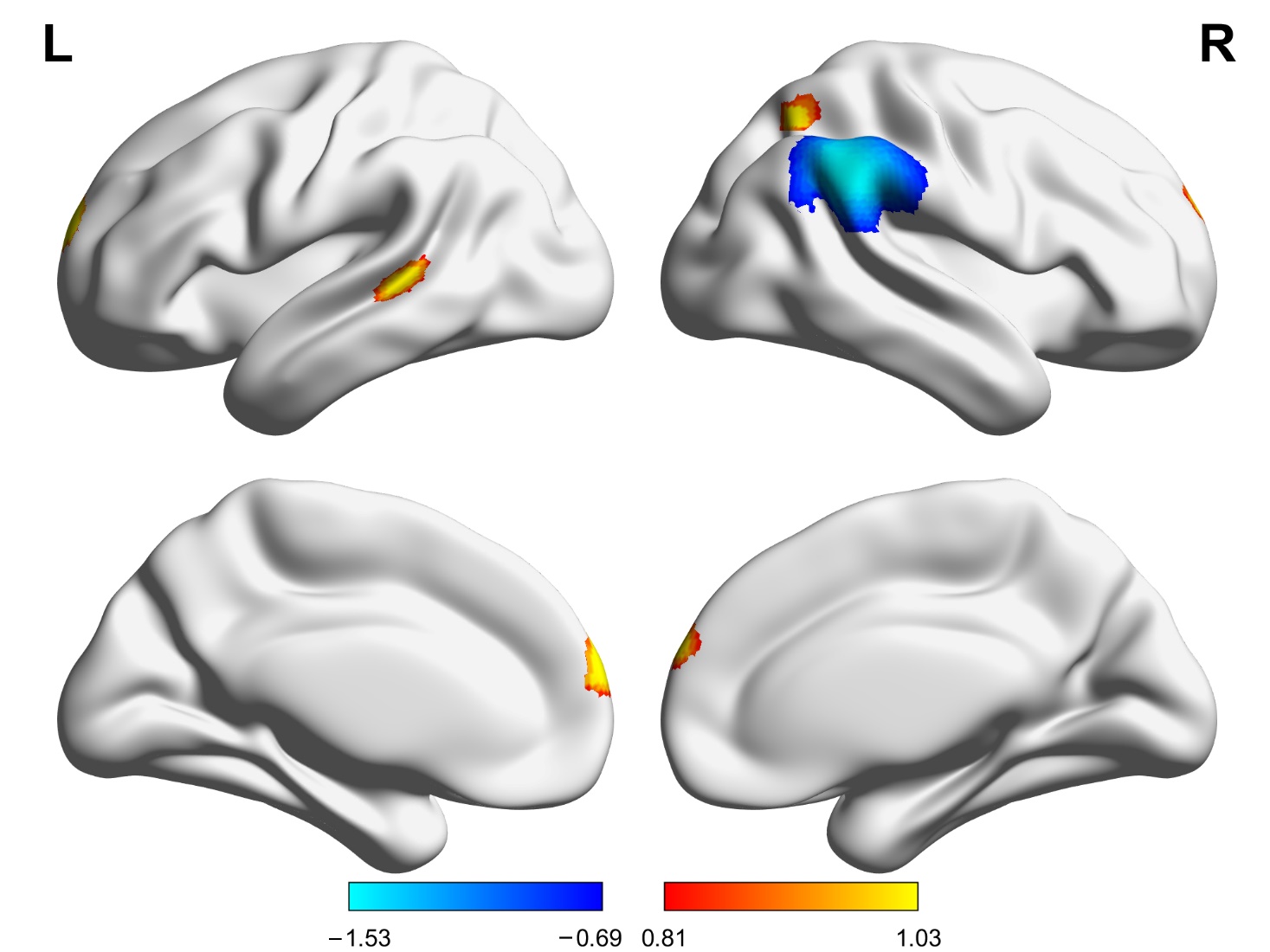


**Figure S2.** Results of cortical thickness differences between ADHD and TDC.


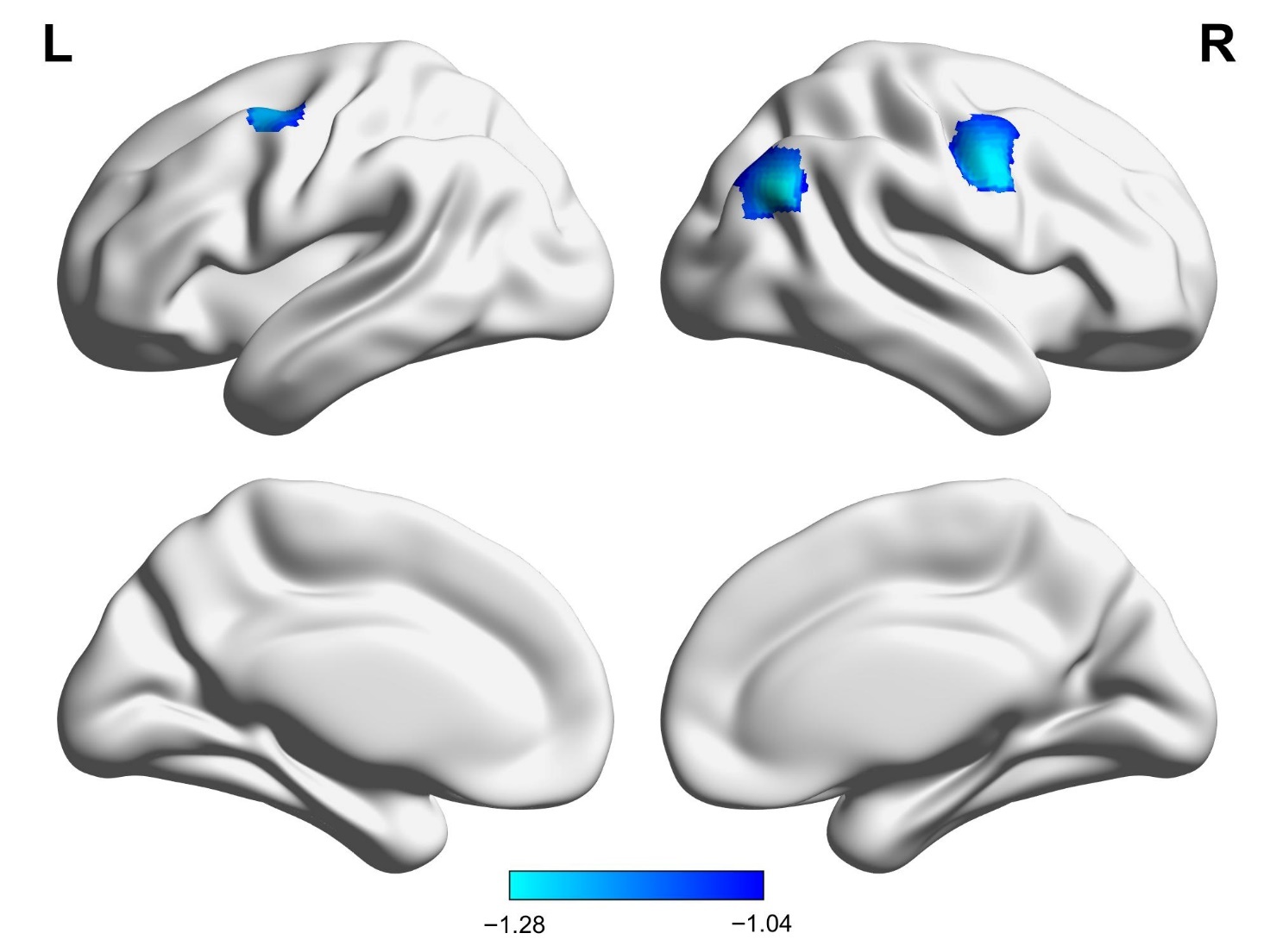

Supplement: Supplementary file 1 — Additional file 1. Supplementary methods of sensitivity, heterogeneity, publication bias, and meta-regression analyses. Table S1. PRISMA 2020 Checklist. Table S2. List of excluded studies that meet other inclusion criteria. Table S3. The checklist of imaging methodology quality assessment for all the articles included in the present meta-analysis. Table S4. Data sources of all public database studies included in the present meta-analysis. Table S5. Differences in cortical thickness between pure ADHD without comorbidity and TDC. Fig. S1. Results of cortical thickness differences between ASD and TDC. Fig. S2. Results of cortical thickness differences between ADHD and TDC. [file 12916_2024_3313_MOESM1_ESM.docx]
